# The Relationship between the Laboratory Biomarkers of SARS-CoV-2 Patients with Type 2 Diabetes at Discharge and the Severity of the Viral Pathology

**DOI:** 10.3390/jpm14060646

**Published:** 2024-06-17

**Authors:** Patricia-Andrada Reștea, Ștefan Țigan, Laura Grațiela Vicaș, Luminita Fritea, Mariana Eugenia Mureșan, Felicia Manole, Daniela Elisabeta Berdea

**Affiliations:** 1 Department of Preclinical Discipline, Doctoral School of Biomedical Science, Faculty of Medicine and Pharmacy, University of Oradea, 1st December Square 10, 410073 Oradea, Romania; 2 Department of Medical Informatics and Biostatistics “Iuliu Hatieganu”, University of Medicine and Pharmacy, 400349 Cluj-Napoca, Romania; stigan@umfcluj.ro; 3 Department of Pharmacy, Faculty of Medicine and Pharmacy, University of Oradea, 1st December Square 10, 410073 Oradea, Romania; 4 Department of Preclinical Discipline, Faculty of Medicine and Pharmacy, University of Oradea, 1st December Square 10, 410073 Oradea, Romania; 5 Department of Surgery, Faculty of Medicine and Pharmacy, University of Oradea, 1st December Square 10, 410073 Oradea, Romania; 6 Department of Morphological Disciplines, Faculty of Medicine and Pharmacy, University of Oradea, 1st December Square 10, 410073 Oradea, Romania; deberdea@uoradea.ro

**Keywords:** SARS-CoV-2, type 2 diabetes, procalcitonin, CRP, ESR, LDH, fibrinogen, discharge, severity

## Abstract

In this study, we evaluated the discharge status of patients with type 2 diabetes mellitus and SARS-CoV-2 infection, focusing on the inflammatory profile through biomarkers such as procalcitonin, CRP, LDH, fibrinogen, ESR, and ferritin, as well as electrolyte levels and the prior diagnosis of diabetes or its identification at the time of hospitalization. We assessed parameters at discharge for 45 patients admitted to the Clinical Hospital “Gavril Curteanu” Oradea between 21 October 2021, and 31 December 2021, randomly selected, having as the main inclusion criteria the positive RT-PCR rapid antigen test for viral infection and the diagnosis of type 2 diabetes. At discharge, patients with type 2 diabetes registered significantly lower mean procalcitonin levels among those who survived compared to those who died from COVID-19. In our study, ferritin and hemoglobin values in individuals with type 2 diabetes were outside the reference range at discharge and correlated with severe or moderate forms of COVID-19 infection. Additionally, elevated ferritin levels at discharge were statistically associated with hypokalemia and elevated levels of ESR at discharge. Another strong statistically significant correlation was identified between high CRP levels at discharge, strongly associated (*p* < 0.001) with elevated LDH and fibrinogen levels in patients with type 2 diabetes and SARS-CoV-2 viral infection. The increase in CRP was inversely statistically associated with the tendency of serum potassium to decrease at discharge in patients with type 2 diabetes and COVID-19. Identifying type 2 diabetes metabolic pathology at the time of hospitalization for SARS-CoV-2 infection, compared to pre-infection diabetes diagnosis, did not significantly influence the laboratory parameter status at the time of discharge. At the discharge of patients with type 2 diabetes and viral infection with the novel coronavirus, procalcitonin was significantly reduced in those who survived COVID-19 infection, and disease severity was significantly correlated with hyperferritinemia and decreased hemoglobin at discharge. Hyperferritinemia in patients with type 2 diabetes and COVID-19 at discharge was associated with hypokalemia and persistent inflammation (quantified by ESR at discharge). The low number of erythrocytes at discharge is associated with maintaining inflammation at discharge (quantified by the ESR value).

## 1. Introduction

In December 2019, the entire world witnessed the emergence of respiratory infection outbreaks and pneumonia in Wuhan, China, caused by a new type of coronavirus initially named 2019-nCoV, later identified as severe acute respiratory syndrome coronavirus 2 (SARS-CoV-2). This virus spread rapidly among humans, leading to the COVID-19 pandemic at the beginning of 2020, with significant medical and socio-economic implications [1]. Over time, two other types of beta-coronaviruses have been implicated in severe respiratory infections transmitted from different animal species to humans: SARS-CoV in 2002 and MERS-CoV in 2012. However, while these viruses belong to the same large family of Coronaviridae, SARS-CoV-2 has had the largest pandemic impact, with the most rapid spread and numerous severe forms and complications affecting populations globally [2,3]. SARS-CoV-2 is a single-stranded RNA virus in the structure of which non-structural and structural proteins have been identified. Among these, a significant role is played by the spike S structural surface protein, which is crucial in the virus’s pathogenesis and entry into cells. Through its S1 subunit, it attaches to receptors on the cell surface, and through S2, it mediates membrane fusion [4,5]. In addition to the spike protein, other structural proteins of the coronavirus include the M protein, important in viral assembly and the most abundant one; the E envelope protein, the smallest one, with three binding domains, playing a role in assembly and virulence; and the nucleocapsid N protein with five RNA-binding domains [6,7,8]. The functional receptor of SARS-CoV-2 to which the spike protein binds is similar to that of SARS-CoV and is represented by the angiotensin-converting enzyme 2 (ACE2), a component of the renin–angiotensin–aldosterone system [9]. As of 3 March 2024, the World Health Organization has reported an impressive number of SARS-CoV-2 infections, specifically 774,834,251 declared cases, with 7,037,007 deaths [10].

There is a wide list of various biomarkers associated with COVID-19 whose concentration depends on the infection severity, being included in 4 big classes: hematological, biochemical, coagulation, and inflammatory biomarkers (Table 1) [11]. In most cases, SARS-CoV-2 leads to multiple organ/system damage; therefore, the analysis of several biomarkers is crucial for dysfunction monitoring. Laboratory biomarkers play a vital role in the diagnosis and prognosis of patients with multiorgan involvement of COVID-19 (respiratory, cardiovascular, neurologic systems, inflammation, coagulation and hemostasis, metabolic function, kidney and liver function), and the testing time is also important [12,13,14].

Chronic inflammation and insulin resistance associated with type 2 diabetes, including the NF-Kappa-B pathway, which plays an essential role in the body’s response to inflammation, are further exacerbated by the SARS-CoV-2 infection. The increase in proinflammatory cytokines and the onset of cytokine storms can lead to multiple organ failures in the context of COVID-19 [15,16]. Procalcitonin is a precursor protein of calcitonin and serves as a marker associated with inflammation, but particularly with septic conditions more frequently, often but not exclusively, linked to bacterial superinfection, playing a role in the severity of COVID-19 [17,18]. C-reactive protein (CRP) is an acute-phase protein synthesized in the liver, serving as a crucial indicator of systemic inflammation. It is associated with severe forms of viral infection with the novel coronavirus, and it can act as an independent factor for mortality depending on the genetic polymorphism of CRP in COVID-19 [19,20,21]. The increase in CRP and procalcitonin levels in patients with diabetes and COVID-19 has been mentioned in the literature as being associated with acute complications of type 2 diabetes, including difficult-to-control hyperglycemia, diabetic ketoacidosis, and hyperosmolar hyperglycemic syndrome [22]. Lactate dehydrogenase (LDH) is an enzyme from the oxidoreductase class that also acts as an acute-phase reactant in systemic inflammation. In COVID-19 infection, LDH has been associated with a negative prognosis and increased severity since the beginning of the pandemic. Its elevation may be linked to organic damage and hypoxia [23]. The increase in LDH as a nonspecific enzyme, starting from the eighth day of SARS-CoV-2 infection, has been reported in studies in the literature, and it has been the most important risk factor for mortality in COVID-19 patients [24]. Furthermore, in COVID-19 infection, LDH levels as a marker of oxidative stress have been shown to correlate with ambient oxygen saturation, anisocytosis, and disease severity [25]. Another acute-phase protein is fibrinogen, the precursor of fibrin, which consists of two subunits with three polypeptide chains of Aalpha, Bbeta, and γ. Levels of fibrinogen variants have been associated with severe forms of SARS-CoV-2 infection [26,27]. This finding was also presented in our previous studies, which included data from patients at the time of admission [28,29]. D-dimers represent a fibrin degradation product, and high levels of d-dimers are associated with prothrombotic risk, lung injury, and severe forms of COVID-19 [30,31]. Ferritin is the major intracellular iron-storage protein, and hyperferritinemia is associated with severe forms of SARS-CoV-2 viral infection, which is an independent factor in mortality [32]. SARS-CoV-2 viral infection causes alteration of hemoglobin, red blood cells, and ESR levels due to oxidative stress and inflammation; hematological parameters are markers of severe forms of viral infection [33]. Also, an imbalance of electrolytes, such as hyponatremia, hypokalemia, or hypocalcemia, may be an independent prognostic factor or severity in COVID-19 [34,35,36].

In the current study, we analyzed the laboratory parameters at the discharge of patients with SARS-CoV-2 infection who also have type 2 diabetes mellitus. The aim of this study was to evaluate biomedical parameters at discharge in individuals with type 2 diabetes and SARS-CoV-2 infection, considering significant deviations from reference biological intervals and the influence of the clinical form of SARS-CoV-2 infection. Another objective of this study was to compare the mean of various biomedical parameters at discharge in individuals with type 2 diabetes and SARS-CoV-2 infection based on pre-existing diabetes or diabetes diagnosed at the time of hospitalization.

## 2. Material and Methods

### 2.1. Study Design

This study was based on 45 patients hospitalized at the Clinical Hospital “Gavril Curteanu” Oradea, the actual Bihor County Emergency Hospital, Coposu location, between 21 October 2021 and 31 December 2021, randomly selected, having as the main inclusion criteria the positive RT-PCR (reverse transcription polymerase chain reaction) rapid antigen test for viral infection and the diagnosis of type 2 diabetes.

This research was conducted in accordance with the Helsinki Declaration, and the protocol was approved by the Ethics Committee of the Clinical Hospital “Gavril Curteanu” Oradea (No. 32652/16 November 2020) and by the Ethics Committee of the University of Oradea (No. 5/A, 21 September 2020). All patients included in this study provided written consent to participate in this research.

The inclusion criteria were SARS-CoV-2 virus infection confirmed by a positive RT-PCR/rapid antigen test and the presence of type 2 diabetes, whether pre-existing or newly diagnosed at the time of hospitalization. The exclusion criteria from the group of patients included the absence of infection with the SARS-CoV-2 virus by negative RT-PCR/rapid antigen test, the absence of type 2 diabetes, or the presence of type 1 diabetes.

### 2.2. Data Collection

The data were collected in an EXCEL file, including the following biomedical parameters at discharge: procalcitonin, CRP, LDH, fibrinogen, ESR (erythrocyte sedimentation rate), ferritin, hemoglobin, erythrocyte count, serum potassium, serum sodium, D-dimers, and the severity of COVID-19 on CT (mild, moderate, and severe forms).

### 2.3. Statistical Analysis

To perform the statistical calculations, the EXCEL data file, which contained the study data, was converted into an SPSS file, and the statistical processing was performed with the statistical software SPSS version 20. Adequate statistical tests were used for the analysis: the Student’s *t*-test for independent samples, the Binomial test and Fisher’s exact test, continuous outcomes performed by the Mann–Whitney *U* test, as well as the non-parametric Spearman. Also, the significance threshold *p* = 0.05 (=5%) was used for the case when the result of the analysis was significant, and *p* = 0.01 was also used for the case when the result of the analysis was strongly significant (*p* < 0.01).

## 3. Results

In this study, we analyzed laboratory tests at the discharge of patients with type 2 diabetes mellitus, whether pre-existing or newly identified at the time of hospitalization, and SARS-CoV-2 infection, based on the severity form on CT (mild, moderate, or severe). We also conducted mean comparisons, correlations between evaluated parameters, and adherence to the reference interval (Table 1).

According to Table 1, the application of the Binomial Test distinguished the following situations:

(i) The proportion of values in the reference range (YES) was strongly statistically significantly higher than those that were not in the reference range (NO) (*p* < 0.001). This occurred for the following parameters at discharge: RBC, Cl^−^, and Na^+^. All values were within the reference range for K^+^ at discharge.
(ii) The proportion of values in the reference range (YES) was statistically significantly lower than those that were not in the reference range (NO) (*p* < 0.05). This occurred for the next parameters at discharge, such as ferritin, CRP, LDH, procalcitonin, fibrinogen, D-dimer, and ESR.
(iii) The proportion of values in the reference range (YES) did not differ statistically significantly from those that were not in the reference range (NO) (*p* ≥ 0.05). This occurred for the parameter Hb at discharge. In cases i and ii, in most situations, it was found that the applied Binomial Test was highly statistically significant, i.e., *p* < 0.001.

In Table 2, by applying the Fisher test to the contingency table, a statistically significant association (*p* = 0.039 < 0.05) was obtained between the fact that ferritin values at discharge were not in the biological reference range and the subject having severe or moderate COVID-19 disease.

In Table 3, by applying the Fisher test to the contingency table, a statistically significant association (*p* = 0.049 < 0.05) was obtained between the fact that hemoglobin values at discharge were outside the biological reference range and the fact that the subject had severe or moderate COVID-19 disease.

From Table 4, based on the Student’s *t*-test for independent samples, the means of the following parameters at discharge: ferritin, Hb, LDH, fibrinogen, ESR, Na^+^, K^+^, and Cl^−^ did not differ significantly statistically between the female and male genders (*p* > 0.05).

Based on the Mann–Whitney non-parametric test (M–W test) for independent samples, Table 5 indicated that the means of the next parameters at discharge (LDH, CRP, procalcitonin, D-dimers, and RBC) did not differ significantly by gender (*p* > 0.05).

Biomedical variables in this study did not significantly differ statistically between urban and rural environments, as observed in Table 6.

Most of the biomedical variables in this study did not statistically significantly differ based on the severity of COVID-19 (a mild, moderate, or severe form of COVID-19). However, statistically significant differences were observed for the following parameters at discharge: *Ferritin*, where the mean for the group of subjects with at most moderate COVID was statistically significantly lower than those with severe COVID (*t*-test, *p* < 0.05); *LDH*, where the mean for the group of subjects with at most moderate COVID was statistically significantly lower than those with severe COVID (*t*-test, *p* < 0.01); and *CRP*, where the mean for the group of subjects with at most moderate COVID was lower than those with severe COVID, although not statistically significant (Table 7).

Statistically significant differences were observed in the next parameters at discharge: ferritin, where the mean for the survivor group was significantly lower than that of the deceased (*t*-test, *p* < 0.05); LDH, where the mean for the survivor group was strongly statistically significantly lower than that of the deceased (*t*-test, *p* < 0.01); and CRP, where the mean for the survivor group was strongly statistically significantly lower than that of the deceased (Mann–Whitney test, *p* < 0.01) (Table 8).

In relation to pre-existing type 2 diabetes at the time of hospitalization for COVID-19 or its diagnosis upon hospitalization (0 = debut of diabetes, 1 = pre-existing diabetes), most of the study’s biomedical variables did not differ statistically significantly on average (Table 9).

RBC at discharge statistically significantly correlated non-parametrically (Spearman) (*p* < 0.05) with both Hb at discharge and ESR at discharge. ESR at discharge showed a statistically significant decreasing trend when RBC at discharge increased (Spearman correlation coefficient Ro < 0) (Table 10).

K^+^ at discharge showed a statistically significant decreasing trend when CRP at discharge increased (Spearman correlation coefficient Ro < 0), while for the other parameters, a statistically significant increase occurred when CRP at discharge increased (Ro > 0). Additionally, strongly statistically significant correlations (*p* < 0.01) were observed between CRP, LDH, fibrinogen, and K at discharge (Table 11).

K^+^ at discharge showed a decreasing trend (statistically significant) when ferritin at discharge increased (Pearson correlation coefficient R < 0), while ESR at discharge showed an increasing trend (statistically significant) when ferritin at discharge increased (R > 0) (Table 12).

The ESR at discharge showed a decreasing trend (statistically significant) when the Hb at discharge increased (Pearson correlation coefficient R < 0), while the LDH at discharge exhibited an increasing trend (statistically significant) when the Hb at discharge increased (R > 0) (Table 13).

## 4. Discussion

In this study, the mean procalcitonin level at discharge was significantly lower in those who survived the viral infection and who were discharged compared to those who died due to SARS-CoV-2. In the literature, an increase in procalcitonin levels in individuals with SARS-CoV-2 infection in the intensive care unit has been associated with an unfavorable prognosis [37]. In another prospective cohort study, an increase in inflammatory parameters was observed in individuals with severe forms of COVID-19 infection [38]. In a retrospective study, the relationship between increased D-dimer levels, hyperferritinemia, and severe forms of infection was highlighted and correlated with transfer to intensive care and the need for intubation and mechanical ventilation [39].

In our study, we analyzed various parameters at discharge and identified an association between ferritin values outside the reference range at discharge and severe or moderate forms of COVID-19 infection in patients with type 2 diabetes. A similar pattern was observed between hemoglobin values at discharge and moderate and severe forms of COVID-19 infection in patients with type 2 diabetes. Ferritin at discharge in type 2 diabetic patients with at most moderate forms of COVID-19 infection was statistically significantly lower (*p* < 0.05) compared to those with severe disease. Studies in the literature have shown higher ferritin levels in individuals with diabetes and SARS-CoV-2 compared to patients without diabetes, and much higher ferritin levels in women compared to men who had the viral infection [40,41].

Another aspect of our study is that the mean ferritin level at discharge in diabetic patients who survived the viral infection was statistically significantly lower than in those who died (*p* < 0.05), and the mean LDH level at discharge was strongly statistically significantly lower in survivors (*p* < 0.001), along with the mean CRP level at discharge (*p* < 0.001). Consistent with our study, data from the literature showed elevated levels of LDH, CRP, fibrinogen, and ferritin as biomarkers of high infectious mortality in the context of the novel coronavirus [42].

Regarding the increased ferritin level at discharge, it was statistically associated with decreased potassium levels at discharge and increased ESR levels at discharge, and the mean ferritin levels at discharge were associated with the severity of COVID-19 infection. Hypokalemia has been mentioned in the literature as a marker of severity and has been associated with intensive care unit admission [43].

Changes in hematological parameters in the context of SARS-CoV-2 infection have been mentioned in certain studies regarding the trend of decreased red blood cell count and hemoglobin levels in patients who have contracted the virus [44]. Faghih Dinevari, M., Somi, M.H., and Sadeghi Majd, E. et al. have described a link between the negative prognosis of SARS-CoV-2 infection in the general population and the presence of anemia in infected patients [45].

In our research, we identified that the LDH value at discharge in individuals with metabolic pathology, such as type 2 diabetes, who had mild and moderate forms of viral infection was significantly lower (*p* < 0.001) than in those patients who had severe forms. CRP at discharge in patients with type 2 diabetes and COVID-19 was lower in those with mild and moderate forms than in those with severe forms on chest CT, however, without statistically significant significance.

Another identified correlation was regarding the high levels of CRP at discharge, strongly statistically associated (*p* < 0.001) with the increase in LDH and fibrinogen levels in patients with type 2 diabetes and SARS-CoV-2 viral infection. On the other hand, the increase in CRP was inversely correlated with the tendency for serum potassium to decrease at discharge in patients with diabetes. The decreasing trend in potassium was also correlated with hyperferritinemia and increased ESR at discharge.

In another comparative study between symptomatic and asymptomatic forms of COVID-19, the levels of ferritin, glucose, CRP, and D-dimers in the context of SARS-CoV-2 infection were described in relation to severe forms of viral pathology [46].

Similarly, we observed that the decreasing trend in ESR was correlated with high levels of hemoglobin at the end of hospitalization. We identified a statistically significant direct correlation between the increase in hemoglobin and the increase in LDH.

Among the parameters involved in iron metabolism, we identified a statistically significant non-parametric correlation between the number of erythrocytes at discharge (RBC) and the value of ESR at discharge in patients with type 2 diabetes and COVID-19 infection, with ESR showing a decreasing trend as the number of erythrocytes increased.

The decreased number of erythrocytes in diabetic patients with SARS-CoV-2 infection has been mentioned in the specialized literature [47,48]. We identified a statistically significant non-parametric correlation at discharge between the number of erythrocytes and the serum hemoglobin level in the patients included in this study.

Some parameters at discharge, such as hemoglobin, the number of erythrocytes, and fibrinogen, as well as the other parameters analyzed, did not show statistically significant differences based on the gender of the diabetic patients included in our study, nor based on their geographical origin.

In a literature study conducted in Heidelberg, Germany, gender differences were mentioned for ferritin, serum iron, and transferrin, which were more pronounced in male patients. However, only serum iron and ferritin could be associated with more severe forms of COVID-19 infection [49].

The detection of diabetes for the first time at the time of hospitalization for COVID-19 infection or the pre-existence of the diagnosis of type 2 diabetes did not represent a statistically significant differentiating factor regarding the values of biological parameters at discharge in the patients from our study. Among the limitations of our study, we mention the small sample size and the fact that the study was conducted at a single center.

## 5. Conclusions

Throughout the hospitalization of individuals with type 2 diabetes, whether pre-existing or diagnosed at the time of admission, and SARS-CoV-2 virus infection, it is important to evaluate laboratory parameters at the time of discharge, considering the severity of the viral infection. This comprehensive approach supports clinicians and, most importantly, patients in clinical, paraclinical, and therapeutic management. In this study, we evaluated laboratory analyses at discharge in individuals with metabolic disorders such as type 2 diabetes and viral infection with the novel coronavirus, focusing on significant deviations from the reference range, clinical form of infection on CT scans, comparisons of means of parameters at discharge, various correlations between parameters, and the timing of diabetes diagnosis.

Thus, the diagnosis of type 2 diabetes at the time of admission or pre-existing diabetes prior to hospitalization did not significantly influence the laboratory results at discharge. However, increased procalcitonin, hyperferritinemia, decreased hemoglobin, and decreased red blood cell count at discharge were statistically significant severity markers in patients with type 2 diabetes. On the other hand, hyperferritinemia at discharge was correlated with changes in electrolytes, specifically a decrease in serum potassium, and persistent inflammation indicated by an elevated ESR at discharge. The decrease in potassium was significantly correlated with another inflammatory marker, namely an increase in C-reactive protein. These values were not influenced by the subjects’ gender or whether they originated from urban or rural environments. The mean of procalcitonin, ferritin, LDH, and CRP levels at discharge were significantly lower in those who survived the viral infection and who were discharged compared to those who died due to the SARS-CoV-2 viral infection.

## Figures and Tables

**Table 1:** The adherence to the reference biological intervals of the parameters at discharge.

Evaluated Parameters		Ref. Interval	N	Obs. Proportion	Tested Proportion	Binomial Test, *p*
Serum ferritin	Group 1	NO	30	0.73	0.27	0.0001
	Group 2	YES	11	0.27	-	-
	Total	-	41	1.00	-	-
Hemoglobin	Group 1	YES	22	0.52	0.48	0.339
	Group 2	NO	20	0.48	-	-
	Total	-	42	1.00	-	-
RBC	Group 1	YES	32	0.78	0.22	0.0001
	Group 2	NO	9	0.22	-	-
	Total	-	41	1.00	-	-
CRP	Group 1	NO	37	0.88	0.12	0.0001
	Group 2	YES	5	0.12	-	-
	Total		42	1.00	-	-
LDH	Group 1	NO	34	0.81	0.19	0.0001
	Group 2	YES	8	0.19	-	-
	Total		42	1.00	-	-
Procalcitonin	Group 1	NO	38	0.90	0.10	0.0001
	Group 2	YES	4	0.10	-	-
	Total		42	1.00	-	-
Fibrinogen	Group 1	NU	29	0.78	0.22	0.0001
	Group 2	DA	8	0.22	-	-
	Total		37	1.00	-	-
D-dimers	Group 1	NO	29	0.76	0.24	0.0001
	Group 2	YES	9	0.24	-	-
	Total		38	1.00	-	-
ESR	Group 1	NO	26	0.67	0.33	0.0001
	Group 2	YES	13	0.33	-	-
	Total		39	1.00	-	-
Na^+^	Group 1	YES	26	0.63	0.50	0.0001
	Group 2	NO	15	0.37	-	-
	Total		41	1.00	-	-
K^+^	Group 1	NO	41	1.00	0.50	0.0001
	Total		41	1.00	-	-
Cl^−^	Group 1	YES	32	0.78	0.22	0.0001
	Group 2	NO	9	0.22	-	-
	Total		41	1.00	-	-

**Table 2:** The association between ferritin at discharge and COVID-19 severity.

Ref. Int.		COVID-19 Severity Forms		Total	Fisher Test	OR		
		Moderate and Severe	Mild		*p*		L.I	LS.
Serum ferritin	YES	29	1	30	0.014	16.571	1.594	172.307
	NO	7	4	11	-	-	-	-
Total		36	5	41	-	-	-	-

OR = 16.571 > 1 (1.594 ≤ OR ≤ 172.307) shows the ratio of those with severe or moderate forms of COVID-19 to those with mild forms among subjects with ferritin levels outside the reference range to the same ratio among subjects with ferritin levels within the reference range.

**Table 3:** The association between Hb levels at discharge and the severity of COVID-19.

Ref. Int.		COVID-19 Severity Forms		Total	F Test	RR		
		Moderate and Severe	Mild		*p*	-	L.I	LS.
Hb	YES	20	0	20	0.049	1.294	1.032	1.623
	NO	17	5	22	-	-	-	-
Total		37	5	42	-	-	-	-

Relative Risk RR = 1.294 > 1 was statistically significant, as indicated by its confidence interval presented in Table 3.

**Table 4:** The comparison of quantitative biomedical parameters at discharge, such as ferritin, Hb, LDH, and fibrinogen by gender.

Gender		N	Mean	Std. Deviation	*t*-Test, *p*
Ferritin	F	26	605.085	615.483	0.080
	M	15	1022.693	867.771	-
Hb	F	26	12.677	1.663	0.157
	M	16	13.556	2.280	-
LDH	F	26	391.413	192.013	0.280
	M	16	489.563	387.699	-
ESR	F	25	47.480	29.158	0.902
	M	14	46.286	28.009	-
Na^+^	F	25	138.101	3.995	0.657
	M	16	137.500	4.514	-
K^+^	F	25	4.204	0.730	0.334
	M	16	3.987	0.631	-
Cl^−^	F	25	101.536	3.643	0.231
	M	16	100.025	4.224	-

**Table 5:** Mann–Whitney non-parametric test (M–W test) for independent samples.

Gender		N	Mean	Std. Deviation.	M–W Test, *p*
LDH	F	26	391.413	192.013	0.856
	M	16	489.563	387.699	-
CRP	F	26	42.875	53.539	0.897
	M	16	81.398	122.251	-
Procalcitonin	F	26	1.100	0.902	0.623
	M	16	2.163	3.465	-
D-dimers	F	24	2324.875	2731.118	0.116
	M	14	1038.929	855.329	-
RBC	F	25	4.440	0.583	0.673
	M	16	4.563	0.629	-

**Table 6:** Comparison of the means of biomedical parameters at discharge by environment (0 = urban, 1 = rural).

Environment		N	Mean	Std. Deviation.	*p*	M–W Test, *p*
Ferritin	0	18	643.606	416.766	0.386	T
	1	23	847.291	912.041	-	-
Hb	0	19	13.121	2.119	0.745	T
	1	23	12.922	1.828	-	-
RBC	0	18	4.444	0.616	0.470	M–W
	1	23	4.522	0.593	-	-
CRP	0	19	63.148	78.737	0.423	M–W
	1	23	52.926	94.835	-	-
LDH	0	19	417.842	339.854	0.822	T
	1	23	437.858	232.439	-	-
Procalcitonin	0	19	1.001	0.693	0.750	M–W
	1	23	1.921	2.971	-	-
Fibrinogen	0	14	477.671	133.024	0.552	T
	1	23	447.639	155.700	-	-
D-dimers	0	15	2120.533	2021.392	0.194	M–W
	1	23	1675.391	2492.591	-	-
ESR	0	17	53.824	33.651	0.194	T
	1	22	41.818	23.006	-	-
Na^+^	0	18	138.200	3.285	0.656	T
	1	23	137.606	4.792	-	-
K^+^	0	18	4.086	0.771	0.789	T
	1	23	4.146	0.643	-	-
Cl^−^	0	18	101.244	4.419	0.671	T
	1	23	100.713	3.527	-	-

**Table 7:** Comparison of the mean values of biomedical parameters at discharge by severity of COVID (1 = mild form, 2 = moderate form, 3 = severe form of COVID).

COVID-19 Severity		N	Mean	Std. Deviation.	*p*	M–W Test, T
Ferritin	Mild and moderate forms	18	518.644	353.389	-	T
	Severe forms	23	945.087	897.280	0.046	-
Hb	Mild and moderate forms	18	13.361	1.384	-	T
	Severe forms	24	12.750	2.268	0.287	-
RBC	Mild and moderate forms	17	4.588	0.507	0.317	M–W
	Severe forms	24	4.417	0.654	-	-
CRP	Mild and moderate forms	18	17.747	21.242	0.213	M–W
	Severe forms	24	87.403	104.873	-	-
LDH	Mild and moderate forms	18	282.667	131.503	0.003	T
	Severe forms	24	538.406	316.578	-	-
Procalcitonin	Mild and moderate forms	18	1.413	2.127	0.258	M–W
	Severe forms	24	1.574	2.418	-	-
Fibrinogen	Mild and moderate forms	16	485.150	121.627	0.350	T
	Severe forms	21	439.081	162.797	-	-
D-dimers	Mild and moderate forms	16	1027.875	1020.499	0.310	M–W
	Severe forms	22	2449.818	2771.788	-	-
ESR	Mild and moderate forms	16	46.063	26.272	0.859	T
	Severe forms	23	47.739	30.329	-	-
Na^+^	Mild and moderate forms	17	137.541	4.690	0.679	T
	Severe forms	24	138.097	3.829	-	-
K^+^	Mild and moderate forms	17	4.344	0.538	0.081	T
	Severe forms	24	3.960	0.756	-	-
Cl^−^	Mild and moderate forms	17	101.194	4.388	0.737	T
	Severe forms	24	100.771	3.603	-	-

**Table 8:** Comparison of the mean of biomedical parameters at discharge by the DEATH parameter (0 = NO, 1 = YES).

Death		N	Mean	Std. Deviation.	*p*	M–W Test, T
Ferritin	NO	33	533.900	363.198	-	T
	YES	8	1681.738	1131.807	0.024	-
Hb	NO	34	12.794	1.791	0.136	T
	YES	8	13.938	2.404	-	-
RBC	NO	33	4.455	0.564	0.656	M–W
	YES	8	4.625	0.744	-	-
CRP	NO	34	24.607	30.276	0.000	M–W
	YES	8	197.561	111.205	-	-
LDH	NO	34	354.375	147.463	0.000	T
	YES	8	745.125	472.891	-	-
Procalcitonin	NO	34	1.156	1.618	0.006	M–W
	YES	8	2.988	3.845	-	-
Fibrinogen	NO	30	439.013	144.791	0.085	T
	YES	7	544.671	129.031	-	-
D-dimers	NO	30	1573.733	1710.071	0.244	M–W
	YES	8	2891.250	3778.140	-	-
ESR	NO	31	45.097	27.285	0.404	T
	YES	8	54.625	33.175	-	-
Na^+^	NO	33	137.580	4.169	0.376	T
	YES	8	139.050	4.175	-	-
K^+^	NO	33	4.199	0.648	0.138	T
	YES	8	3.791	0.820	-	-
Cl^−^	NO	33	100.758	3.522	0.536	T
	YES	8	101.725	5.419	-	-

**Table 9:** Comparison of the means of biomedical parameters at discharge by the parameter of pre-existing type 2 diabetes (0 = debut of diabetes, 1 = pre-existing diabetes).

DM		N	Mean	Std. Dev.	*p*	M–W Test, T
Ferritin	0	5	720.340	449.686	0.905	T
	1	36	763.081	771.915	-	-
Hb	0	6	13.733	1.759	0.332	T
	1	36	12.892	1.969	-	-
RBC	0	6	4.715	0.600	0.206	M–W
	1	35	4.360	0.565	-	-
CRP	0	6	29.677	40.580	0.661	M–W
	1	36	62.196	92.097	-	-
LDH	0	6	361.000	115.864	0.532	T
	1	36	440.104	301.069	-	-
Procalcitonin	0	6	0.958	0.829	0.746	M–W
	1	36	1.596	2.427	-	-
Fibrinogen	0	5	359.160	185.273	0.102	T
	1	32	474.603	136.345	-	-
D-dimers	0	5	1094.800	944.756	0.479	M–W
	1	33	1965.697	2429.159	-	-
ESR	0	5	34.800	15.723	0.308	T
	1	34	48.853	29.525	-	-
Na^+^	0	6	135.200	3.863	0.089	T
	1	35	138.324	4.086	-	-
K^+^	0	6	4.235	0.480	0.665	T
	1	35	4.100	0.728	-	-
Cl^−^	0	6	98.717	3.531	0.131	T
	1	35	101.329	3.877	-	-

**Table 10:** Non-parametric correlations between the level of RBC at discharge and the quantitative parameters of the study at discharge.

	RBC		
	Spearman Ro	*p*	N
Hb	0.897	0.000	41
ESR	−0.506	0.001	38

**Table 11:** Non-parametric correlations between the level of CRP at discharge and the quantitative parameters of the study at discharge.

	CRP		
	Spearman Ro	*p*	N
Ferritin	0.385	0.013	41
LDH	0.549	0.000	42
Procalcitonin	0.311	0.045	42
Fibrinogen	0.448	0.005	37
D-dimers	0.433	0.007	38
K^+^	−0.479	0.002	41

**Table 12:** Pearson parametric correlations between ferritin at discharge and other parameters at discharge.

	Ferritin		
	Pearson R	*p*	N
ESR	0.326	0.043	39
K^+^	−0.364	0.021	40

**Table 13:** Parametric correlations (Pearson) between the parameter Hb at discharge and other parameters at discharge.

	Hb		
	Pearson R	*p*	N
LDH	0.423	0.005	42
ESR	−0.454	0.004	39

## Data Availability

Data are contained with in the article.

## Abbreviations

In this manuscript, the following abbreviations are used:

SARS-CoV-2	Severe acute respiratory syndrome coronavirus 2
COVID-19	Coronavirus disease
ACE2	Angiotensin-converting enzyme 2
CRP	C reactive protein
RT-PCR	Reverse transcription polymerase chain reaction
RBC	Red blood cell
K	Potassium (Kalium)
Cl	Chlorine
Na	Sodium
Hb	Hemoglobin
CT	Computed tomography
LDL	Low density cholesterol
ESR	Erythrocyte sedimentation rate

## References

[1] Zhou P., Yang X.-L., Wang X.-G., Hu B., Zhang L., Zhang W., Si H.-R., Zhu Y., Li B., Huang C.-L. (2020). A pneumonia outbreak associated with a new coronavirus of probable bat origin. Nature.

[2] La Montagne J.R., Simonsen L., Taylor R.J., Turnbull J. (2024). SARS Research Working Group Co-Chairs, Severe Acute Respiratory Syndrome: Developing a Research Response. J. Infect. Dis..

[3] Who Mers-Cov Research Group (2013). State of Knowledge and Data Gaps of Middle East Respiratory Syndrome Coronavirus (MERS-CoV) in Humans. PLoS Curr..

[4] Cao C., Cai Z., Xiao X., Rao J., Chen J., Hu N., Yang M., Xing X., Wang Y., Li M. (2021). Arhitectura genomului ARN SARS-CoV-2 în interiorul virionului. Nat. Commun..

[5] Tang X., Qian Z., Lu X., Lu J. (2023). Adaptive Evolution of the Spike Protein in Coronaviruses. Mol. Biol. Evol..

[6] Zhang Z., Nomura N., Muramoto Y., Ekimoto T., Uemura T., Liu K., Yui M., Kono N., Aoki J., Ikeguchi M. (2022). Structure of SARS-CoV-2 membrane protein essential for virus assembly. Nat. Commun..

[7] Cubuk J., Alston J.J., Incicco J.J., Singh S., Stuchell-Brereton M.D., Ward M.D., Zimmerman M.I., Vithani N., Griffith D., Wagoner J.A. (2021). The SARS-CoV-2 nucleocapsid protein is dynamic, disordered, and phase separates with RNA. Nat. Commun..

[8] Javorsky A., Humbert P.O., Kvansakul M. (2021). Structural basis of coronavirus E protein interactions with human PALS1 PDZ domain. Commun. Biol..

[9] Lan J., Ge J., Yu J., Shan S., Zhou H., Fan S., Zhang Q., Shi X., Wang Q., Zhang L. (2020). Structure of the SARS-CoV-2 spike receptor-binding domain bound to the ACE2 receptor. Nature.

[10] WHO. https://data.who.int/dashboards/covid19/cases?n=c.

[11] Ponti G., Maccaferri M., Ruini C., Tomasi A., Ozben T. (2020). Biomarkers associated with COVID-19 disease progression. Crit. Rev. Clin. Lab. Sci..

[12] Pal M., Muinao T., Parihar A., Roy D.K., Boruah H.P.D., Mahindroo N., Khan R. (2022). Biosensors based detection of novel biomarkers associated with COVID-19: Current progress and future promise. Biosens. Bioelectron. X.

[13] Battaglini D., Lopes-Pacheco M., Castro-Faria-Neto H.C., Pelosi P., Rocco P.R.M. (2022). Laboratory Biomarkers for Diagnosis and Prognosis in COVID-19. Front. Immunol..

[14] Samprathi M., Jayashree M. (2021). Biomarkers in COVID-19: An Up-To-Date Review. Front. Pediatr..

[15] Mozafari N., Azadi S., Mehdi-Alamdarlou S., Ashrafi H., Azadi A. (2020). Inflammation: A bridge between diabetes and COVID-19, and possible management with sitagliptin. Med. Hypotheses.

[16] Ho G., Ali A., Takamatsu Y., Wada R., Masliah E., Hashimoto M. (2021). Diabetes, inflammation, and the adiponectin paradox: Therapeutic targets in SARS-CoV-2. Drug Discov. Today.

[17] Bajić D., Matijašević J., Andrijević L., Zarić B., Lalić-Popović M., Andrijević I., Todorović N., Mihajlović A., Tapavički B., Ostojić J. (2023). Prognostic Role of Monocyte Distribution Width, CRP, Procalcitonin and Lactate as Sepsis Biomarkers in Critically Ill COVID-19 Patients. J. Clin. Med..

[18] Hu R., Han C., Pei S., Yin M., Chen X. (2020). Procalcitonin levels in COVID-19 patients. Int. J. Antimicrob. Agents.

[19] Gebrecherkos T., Challa F., Tasew G., Gessesse Z., Kiros Y., Gebreegziabxier A., Abdulkader M., Desta A.A., Atsbaha A.H., Tollera G. (2023). Prognostic Value of C-Reactive Protein in SARS-CoV-2 Infection: A Simplified Biomarker of COVID-19 Severity in Northern Ethiopia. Infect. Drug Resist..

[20] Fritea L., Fritea L., Sipponen M., Sipponen M., Antonescu A., Antonescu A., Miere F.G., Miere F.G., Chirla R., Chirla R. (2022). Relationship between Pre-Existing Conditions in Covid-19 Patients and Inflammation. Pharmacophore.

[21] Mofrad S.S., Amnieh S.B., Pakzad M.R., Zardadi M., Jajin M.G., Anvari E., Moghaddam S., Fateh A. (2024). The death rate of COVID-19 infection in different SARS-CoV-2 variants was related to C-reactive protein gene polymorphisms. Sci. Rep..

[22] Tao L.-C., Shu H., Wang Y., Hou Q., Li J.-J., Huang X.-L., Hua F. (2024). Inflammatory biomarkers predict higher risk of hyperglycemic crises but not outcomes in diabetic patients with COVID-19. Front. Endocrinol..

[23] Shokr H., Marwah M.K., Siddiqi H., Wandroo F., Sanchez-Aranguren L., Ahmad S., Wang K., Marwah S. (2023). Lactate Dehydrogenase/Albumin To-Urea Ratio: A Novel Prognostic Maker for Fatal Clinical Complications in Patients with COVID-19 Infection. J. Clin. Med..

[24] Nakakubo S., Unoki Y., Kitajima K., Terada M., Gatanaga H., Ohmagari N., Yokota I., Konno S. (2023). Serum Lactate Dehydrogenase Level One Week after Admission Is the Strongest Predictor of Prognosis of COVID-19: A Large Observational Study Using the COVID-19 Registry Japan. Viruses.

[25] Alonso-Bernáldez M., Cuevas-Sierra A., Micó V., Higuera-Gómez A., Ramos-Lopez O., Daimiel L., Dávalos A., Martínez-Urbistondo M., Moreno-Torres V., Ramirez de Molina A. (2023). An Interplay between Oxidative Stress (Lactate Dehydrogenase) and Inflammation (Anisocytosis) Mediates COVID-19 Severity Defined by Routine Clinical Markers. Antioxidants.

[26] de Vries J.J., Visser C., van Ommen M., Rokx C., van Nood E., van Gorp E.C.M., Goeijenbier M., Akker J.P.C.v.D., Endeman H., Rijken D.C. (2023). Levels of Fibrinogen Variants Are Altered in Severe COVID-19. TH Open.

[27] Risman R.A., Belcher H.A., Ramanujam R.K., Weisel J.W., Hudson N.E., Tutwiler V. (2024). Comprehensive Analysis of the Role of Fibrinogen and Thrombin in Clot Formation and Structure for Plasma and Purified Fibrinogen. Biomolecules.

[28] Restea P.-A., Muresan M., Voicu A., Jurca T., Pallag A., Marian E., Vicaș L.G., Jeican I.I., Crivii C.-B. (2023). Antidiabetic Treatment before Hospitalization and Admission Parameters in Patients with Type 2 Diabetes, Obesity, and SARS-CoV-2 Viral Infection. J. Pers. Med..

[29] Reștea P.-A., Țigan Ș., Vicaș L.G., Fritea L., Marian E., Jurca T., Pallag A., Mureșan I.L., Moisa C., Micle O. (2023). Serum Level of Ceruloplasmin, Angiotensin-Converting Enzyme and Transferrin as Markers of Severity in SARS-CoV-2 Infection in Patients with Type 2 Diabetes. Microbiol. Res..

[30] Trimaille A., Thachil J., Marchandot B., Curtiaud A., Leonard-Lorant I., Carmona A., Matsushita K., Sato C., Sattler L., Grunebaum L. (2021). D-Dimers Level as a Possible Marker of Extravascular Fibrinolysis in COVID-19 Patients. J. Clin. Med..

[31] Qeadan F., Tingey B., Gu L.Y., Packard A.H., Erdei E., Saeed A.I. (2021). Prognostic Values of Serum Ferritin and D-Dimer Trajectory in Patients with COVID-19. Viruses.

[32] Kurian S.J., Mathews S.P., Paul A., Viswam S.K., Nagri S.K., Miraj S.S., Karanth S. (2023). Association of serum ferritin with severity and clinical outcome in COVID-19 patients: An observational study in a tertiary healthcare facility. Clin. Epidemiol. Glob. Health.

[33] Russo A., Tellone E., Barreca D., Ficarra S., Laganà G. (2022). Implication of COVID-19 on Erythrocytes Functionality: Red Blood Cell Biochemical Implications and Morpho-Functional Aspects. Int. J. Mol. Sci..

[34] Berni A., Malandrino D., Corona G., Maggi M., Parenti G., Fibbi B., Poggesi L., Bartoloni A., Lavorini F., Fanelli A. (2021). Serum sodium alterations in SARS CoV-2 (COVID-19) infection: Impact on patient outcome. Eur. J. Endocrinol..

[35] Alfano G., Ferrari A., Fontana F., Perrone R., Mori G., Ascione E., Magistroni R., Venturi G., Pederzoli S., Margiotta G. (2021). Hypokalemia in Patients with COVID-19. Clin. Exp. Nephrol..

[36] Reștea P.-A., Tigan Ș., Fritea L., Vicaș L.G., Marian E., Mureșan M.E., Stefan L. (2024). Serum Calcium and Magnesium Levels in Patients with Type 2 Diabetes and COVID-19 Infection Requiring Hospitalization—Correlations with Various Parameters. Microbiol. Res..

[37] Ince F.M., Alkan Bilik O., Ince H. (2024). Evaluating Mortality Predictors in COVID-19 Intensive Care Unit Patients: Insights into Age, Procalcitonin, Neutrophil-to-Lymphocyte Ratio, Platelet-to-Lymphocyte Ratio, and Ferritin Lactate Index. Diagnostics.

[38] Trofin F., Nastase E.V., Roșu M.F., Bădescu A.C., Buzilă E.R., Miftode E.G., Manciuc D.C., Dorneanu O.S. (2023). Inflammatory Response in COVID-19 Depending on the Severity of the Disease and the Vaccination Status. Int. J. Mol. Sci..

[39] Hakami A., Altubayqi T., Qadah E.A., Zogel B., Alfaifi S.M., Refaei E., Sayed A., Alhazmi L., Sayegh M., Alamer A. (2023). Biochemical Analysis of Ferritin and D-dimer in COVID-19 Survivors and Non-survivors. Cureus.

[40] Madfoon Z., Mezher M., Madfoon S. (2022). Comparison of Serum Ferritin Levels between Diabetic Patients with COVID-19 and Non-Diabetic Patients with COVID-19 Based on Age Groups and Gender. Iran. J. War Public Health.

[41] Liani F.N., Mudjanarko S.W., Novida H. (2022). The role of serum ferritin level and disease severity in COVID-19 with type 2 diabetes mellitus patients. Bali Med. J..

[42] Casillas N., Ramón A., Torres A.M., Blasco P., Mateo J. (2023). Predictive Model for Mortality in Severe COVID-19 Patients across the Six Pandemic Waves. Viruses.

[43] Moreno-Pérez O., Leon-Ramirez J.-M., Fuertes-Kenneally L., Perdiguero M., Andres M., Garcia-Navarro M., Ruiz-Torregrosa P., Boix V., Gil J., Merino E. (2020). Hypokalemia as a sensitive biomarker of disease severity and the requirement for invasive mechanical ventilation requirement in COVID-19 pneumonia: A case series of 306 Mediterranean patients. Int. J. Infect. Dis..

[44] Urbano M., Costa E., Geraldes C. (2022). Hematological changes in SARS-COV-2 positive patients. Hematol. Transfus. Cell Ther..

[45] Dinevari M.F., Somi M.H., Majd E.S., Farhangi M.A., Nikniaz Z. (2021). Anemia predicts poor outcomes of COVID-19 in hospitalized patients: A prospective study in Iran. BMC Infect Dis..

[46] Pérez-García N., García-González J., Requena-Mullor M., Rodríguez-Maresca M.Á., Alarcón-Rodríguez R. (2022). Comparison of Analytical Values D-Dimer, Glucose, Ferritin and C-Reactive Protein of Symptomatic and Asymptomatic COVID-19 Patients. Int. J. Environ. Res. Public Health.

[47] Akbariqomi M., Hosseini M.S., Rashidiani J., Sedighian H., Biganeh H., Heidari R., Moghaddam M.M., Farnoosh G., Kooshki H. (2020). Clinical characteristics and outcome of hospitalized COVID-19 patients with diabetes: A single-center, retrospective study in Iran. Diabetes Res. Clin. Pract..

[48] Gavkare A.M., Nanaware N., Rayate A.S., Mumbre S., Nagoba B.S. (2022). COVID-19 associated diabetes mellitus: A review. World J. Diabetes.

[49] Hippchen T., Altamura S., Muckenthaler M.U., Merle U. (2020). Hypoferremia is Associated with Increased Hospitalization and Oxygen Demand in COVID-19 Patients. Hemasphere.

